# Crystal Engineering of Ionic Cocrystals Sustained by the Phenol–Phenolate Supramolecular Heterosynthon

**DOI:** 10.1021/acs.cgd.2c00471

**Published:** 2022-06-21

**Authors:** Shasha Jin, Rana Sanii, Bai-Qiao Song, Michael J. Zaworotko

**Affiliations:** Department of Chemical Sciences and Bernal Institute, University of Limerick, Co., Limerick V94 T9PX, Ireland

## Abstract

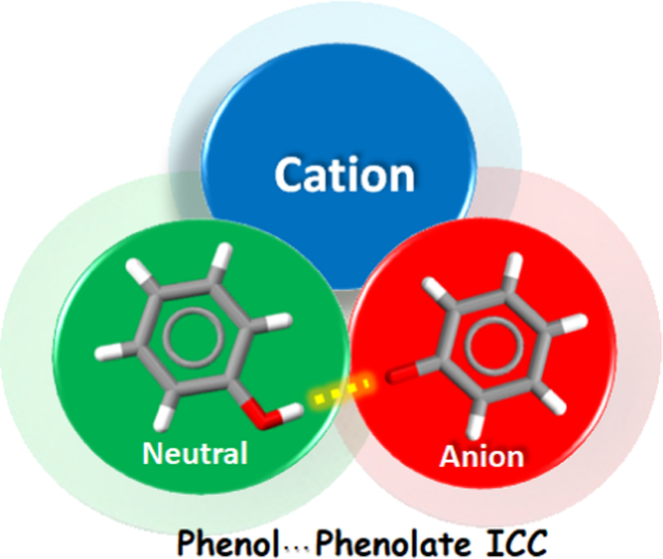

Although crystal engineering strategies are generally well explored in the context of multicomponent crystals (cocrystals) formed by neutral coformers (molecular cocrystals), cocrystals comprised of one or more salts (ionic cocrystals, ICCs) are understudied. We herein address the design, preparation, and structural characterization of ICCs formed by phenolic moieties, a common group in natural products and drug molecules. Organic and inorganic bases were reacted with the following phenolic coformers: phenol, resorcinol, phloroglucinol, 4-methoxyphenol, and 4-isopropylphenol. Nine ICCs were crystallized, each of them sustained by the phenol–phenolate supramolecular heterosynthon (PhOH···PhO^–^). Such ICCs are of potential utility, and there are numerous examples of phenolic compounds that are biologically active, some of which suffer from low aqueous solubility. The propensity to form ICCs sustained by the PhOH···PhO^–^ supramolecular heterosynthon was evaluated through a combination of Cambridge Structural Database (CSD) mining, structural characterization of nine novel ICCs, and calculation of interaction energies. Our analysis of these 9 ICCs and the 41 relevant entries archived in the CSD revealed that phenol groups can reliably form ICCs through charge-assisted PhOH···PhO^–^ interactions. This conclusion is supported by hydrogen-bond strength calculations derived from CrystalExplorer that reveal the PhOH···PhO^–^ interaction to be around 3 times stronger than the phenol–phenol hydrogen bond. The PhOH···PhO^–^ supramolecular heterosynthon could therefore enable crystal engineering studies of a large number of phenolic pharmaceutical and nutraceutical compounds with their conjugate bases.

## Abstract 2

Cambridge Structural Database mining and experimental studies of model compounds reveal that ionic cocrystals sustained by phenol···phenolate hydrogen bonds are formed reliably

## Introduction

The term crystal engineering, coined by Pepinsky in 1955 in the context of metal complexes,^1^ has developed continuously since first implemented by Schmidt’s group in the context of photochemical reactions in the 1960s.^2^ Although in the 1990s the focus of crystal engineering was the design of crystal structures through intermolecular interactions^3^ or coordination bonds, the focus has now evolved to encompass structure–property relationships and applications.^4^ Crystal engineering has thereby been utilized across a broad range of materials including nonlinear optical materials (NLO),^5,6^ organic semiconductors,^7,8^ energetic materials,^9,10^ and metal–organic frameworks.^11^ The most significant application of crystal engineering is perhaps in pharmaceutical science^12−15^ as the functional groups present in active pharmaceutical ingredients (APIs), especially the hydrogen-bonding functional groups that drive biological activity, are well-studied targets for crystal engineering.

Certain intermolecular interactions, such as neutral and charge-assisted hydrogen bonds, and coordination bonds, are amenable to crystal engineering studies thanks to their relatively high strength, predictability, ubiquity, and directionality.^16^ Intermolecular interactions have been classified by motifs and synthons. Etter systematically classified common hydrogen-bonding motifs using “graph set” notation *G*_*d*_^a^(*r*), where *G* is the pattern designator that could be C (chain), R (ring), and D (dimer or other finite set); a represents the number of acceptors; *d* is the number of donors; and *r* is the number of atoms in the repeat unit.^17,18^ Graph set theory remains a valuable tool for researchers to compare structures to this day. Shortly after, Desiraju introduced the term “supramolecular synthon” to represent a structural unit or building block in crystal structures.^19^ Supramolecular synthons were subclassified by us in 2003 into supramolecular homosynthons, between the same functional groups (e.g., carboxylic acid···carboxylic acid dimers) and supramolecular heterosynthons and between different but complementary functional groups (e.g., carboxylic acid···amide supramolecular heterosynthon).^14^ The understanding of supramolecular synthons, their geometries, and their frequency of occurrence in the Cambridge Structural Database (CSD) is key to the rational design of novel multicomponent crystal forms such as cocrystals and hydrates.^20^

Cocrystals are solids that are crystalline single-phase materials comprised of two or more different molecular and/or ionic compounds generally in a stoichiometric ratio that are neither simple salts nor solvates.^21^ Cocrystals are of interest in part because they can be readily accessible and amenable to crystal engineering. Cocrystals include molecular cocrystals^22^ (MCCs), which are comprised of two or more nonvolatile neutral molecules (coformers), and ionic cocrystals^23^ (ICCs), which involve at least one coformer that is a salt. ICCs are necessarily sustained by charge-assisted hydrogen bonds or, if metal ions are involved, coordination bonds.^24,25^ ICCs are therefore comprised of at least three components, a cation, an anion, and an additional molecule or salt. Given that MCCs of a compound typically offer a single variable, the coformer, ICCs can exhibit greater diversity in terms of composition and physicochemical properties compared to MCCs.^4^ Additionally, if an ICC is formed in which one coformer is an API and another is an API salt, then this type of ICC provides an opportunity to generate low-dosage solid forms since the biologically active component(s) represent most of the mass of the ICC. Although ICCs are generally less studied than MCCs, the first cocrystal involving sodium chloride and urea was an ICC^26^ and, to our knowledge, at least five ICCs have been selected and developed for use in marketed drug products: Depakote (valproate sodium and valproic acid)^27^ in 1983; Lexapro (escitalopram oxalate salt and oxalic acid)^28^ in 2002; Odomzo (the salt of sonidegib with phosphate and phosphoric acid)^29^ in 2015; Entresto (valsartan and sacubitril)^30^ in 2015; Seglentis (celecoxib and tramadol hydrochloride)^31^ in 2021.

Phenolic drug molecules and nutraceutical compounds are of topical interest because they can exhibit biological effects such as antioxidant and anti-inflammatory activity, inhibit lipid peroxidation initiated in rat brain homogenates by Fe^2+^ and l-ascorbic acid, and inhibit the formation of oxygen free radicals.^32,33^ Statistically, ca. 8.7% of approved small-molecule drugs contained in DrugBank Online (Version 5.1.9.)^34^ are phenolic drugs, and phenol groups are present in ca. 10.1% of single-component biologically active compounds archived in the CSD (Scheme S1). However, many such compounds exhibit low aqueous solubility and would therefore be classified as BCS class II.^35^ Their efficacy would therefore be hindered and an otherwise promising drug molecule could be rendered unsuitable for use in an orally delivered drug product.^36,37^ Molecular cocrystals are now well known to modulate the physicochemical properties of phenolic compounds including solubility,^38^ bioavailability,^39^ stability,^40^ and mechanical properties^41^ while preserving their inherent biological activity.

With respect to crystal engineering, phenols offer medium hydrogen-bond donor strength and are established as being able to form supramolecular heterosynthons with hydrogen-bond acceptors such as chloride anions,^20^ carboxylate moieties,^42^ and aromatic nitrogen bases.^43^ Conversely, the phenol–phenolate (PhOH···PhO^–^) interaction is considered to be a strong hydrogen bond. In solution, PhOH···PhO^–^ systems known as homoconjugated complexes readily form as measured by their conjugated constants.^44^ PhOH···PhO^–^ complexes can have a negative impact on titration experiments and play an important role in enzyme catalysis.^45^ Buytendyk et al. reported the PhOH···PhO^–^ interaction energy in the gas phase to be as high as 26–30 kcal/mol, 60% of that of the HF···F^–^ interaction, the strongest hydrogen bond known.^46^ Crystal structures involving PhOH···PhO^–^ interactions archived in the CSD were mainly studied for structural insight^47^ or applications such as organic synthesis (Kolbe–Schmitt synthesis)^48,49^ and NLO.^50^ As such, there remains a lack of systematic crystal engineering studies on ICCs containing the PhOH···PhO^–^ supramolecular heterosynthon.

**Chart 1:**
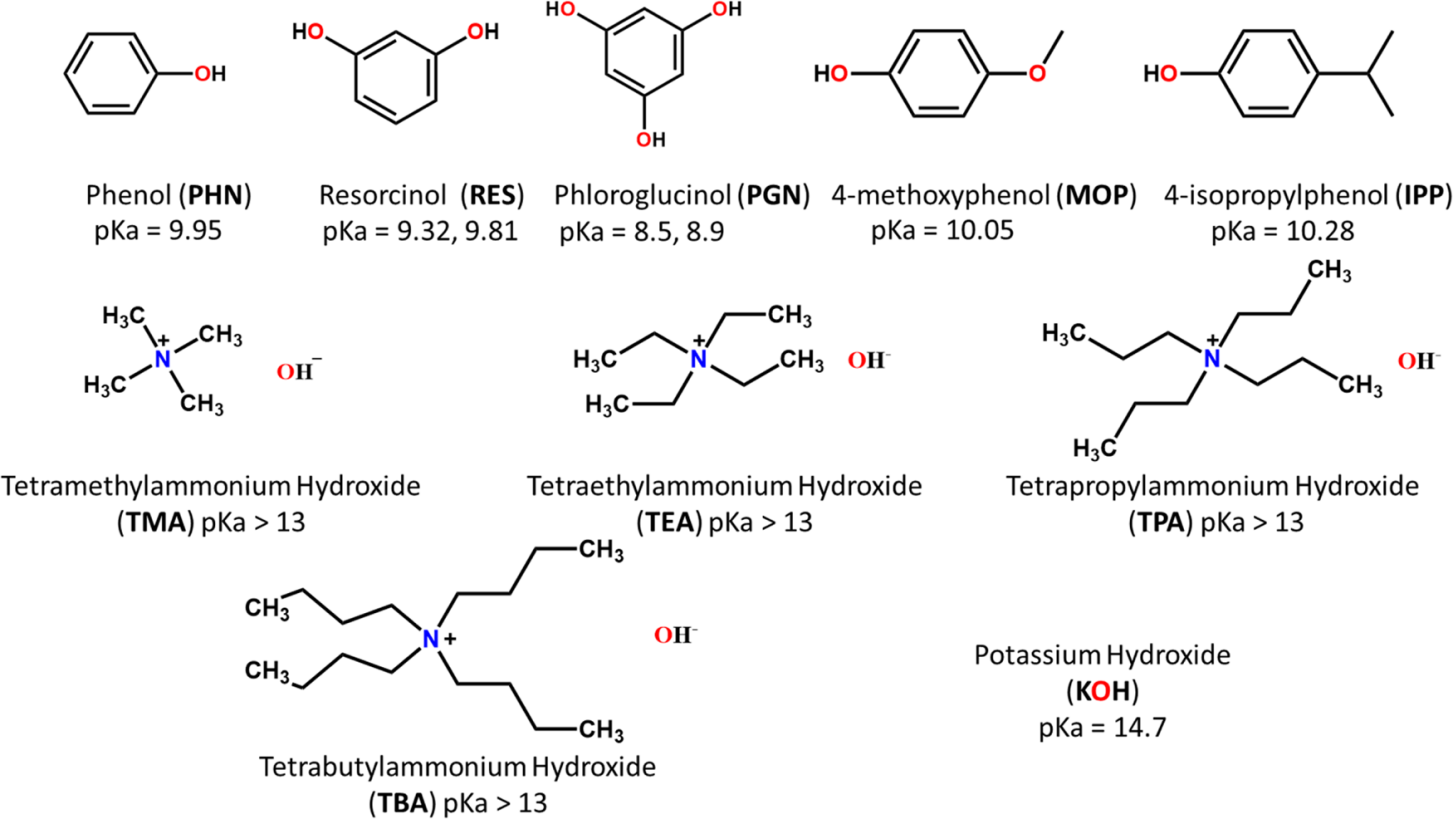
Molecular Structures and 3-Letter Abbreviations of Coformers Used Herein

In this contribution, we address ICC formation involving phenols through a systematic CSD and experimental study that explores PhOH···PhO^–^ supramolecular heterosynthons. Specifically, phenol and four other substituted phenol derivatives were selected as model compounds and reacted with tetraalkylammonium hydroxides and potassium hydroxide (Chart 1). In addition, the hydrogen-bond strength of PhOH···PhO^–^ and phenol–phenol (PhOH···PhOH) interactions in the obtained ICCs were examined using CrystalExplorer.

## Experimental Section

A library of cocrystal formers containing five phenol derivatives, four organic bases, and one inorganic base was chosen for this study (Chart 1). Cocrystallization reactions afforded the following nine ionic cocrystals: phenol·phenolate·tetramethylammonium, **PHNTMA**; phenol·phenolate·potassium, **PHNKOH**; 1,3,5-benzentriol·1,3,5-benzentriolate·tetramethylammonium, **PGNTMA**; 1,3,5-benzentriol·1,3,5-benzentriolate·tetraethylammonium, **PGNTEA**; 4-isopropylphenol·4-isopropylphenolate·potassium, **IPPKOH**; 4-isopropylphenol·4-isopropylphenolate·tetrapropylammonium, **IPPTPA**; 4-isopropylphenol·4-isopropylphenolate·tetrabutylammonium, **IPPTBA**; 4-methoxyphenol·4-methoxyphenolate·tetrabutylammonium, **MOPTBA**; and resorcinol·resorcinolate·tetramethylammonium, **RESTMA**.

### Synthesis

All reagents and chemicals were purchased from Sigma-Aldrich and used without further purification. Single crystals of ICCs were obtained via slow evaporation of stoichiometric amounts of starting materials in a range of solvents at room temperature and harvested from solution before complete evaporation of their mother liquors had occurred.

### PHNTMA

PHN (940 mg, 10 mmol) and TMA (2.7 mL, 5.94 mmol, 2.2 M in methanol) were dissolved in 10 mL of acetonitrile (MeCN). The solvent was evaporated under vacuum until a viscous liquid was obtained. Colorless crystals were harvested after exposing the viscous liquid to ambient conditions overnight.

### PHNKOH

PHN (9.4 mg, 0.1 mmol) and KOH (0.1 mL, 0.1 mmol, 1 M in water) were dissolved in 1 mL of methanol (MeOH). The solution was slowly evaporated at room temperature, and colorless crystals were obtained after 1 day.

### PGNTMA

PGN (25.2 mg, 0.2 mmol) and TMA (0.045 mL, 0.1 mmol, 2.2 M in MeOH) were dissolved in 1 mL of MeOH. The solution was slowly evaporated at room temperature, and colorless crystals were obtained after 1 day.

### PGNTEA

PGN (25.2 mg, 0.2 mmol) and TEA (0.021 mL, 0.1 mmol, 4.8 M in MeOH) were dissolved in 1 mL of MeOH. The solution was slowly evaporated at room temperature, and colorless crystals were obtained after several hours.

### IPPKOH

IPP (27.2 mg, 0.2 mmol) and KOH (0.4 mL, 0.1 mmol, 0.25 M in H_2_O) were dissolved in 1 mL of MeOH. The solution was slowly evaporated at room temperature, and colorless crystals were obtained after 1 day.

### IPPTPA

IPP (27.2 mg, 0.2 mmol) and TPA (0.1 mL, 0.1 mmol, 1 M in H_2_O) were dissolved in 1 mL of MeCN. The solution was slowly evaporated at room temperature, and colorless crystals were obtained after 1 day.

### IPPTBA

IPP (136.2 mg, 1 mmol) and TBA (0.33 mL, 0.5 mmol, 1.5 M in H_2_O) were slurried in 1 mL of H_2_O overnight. The undissolved solid was isolated by filtration and dried in an oven. Colorless single crystals were obtained by dissolving the solid in MeOH, followed by slow evaporation at room temperature.

### MOPTBA

MOP (248.2 mg, 2 mmol) and TBA (0.67 mL, 1 mmol, 1.5 M in water) were slurried in 2 mL of H_2_O for 24 h. The undissolved solid was isolated by filtration and dried in an oven. Colorless single crystals were obtained by dissolving the solid in acetone (ACE), followed by slow evaporation at room temperature.

### RESTMA

RES (36.6 mg, 0.33 mmol), IPP (9 mg, 0.07 mmol), and TMA (0.09 mL, 0.2 mmol, 2.2 M in MeOH) were dissolved in 1 mL of MeOH. The solution was slowly evaporated at room temperature, and colorless crystals were obtained after 1 week.

### Powder X-ray Diffraction (PXRD)

All PXRD data were collected on an Empyrean diffractometer (PANalytical) with the following experimental parameters: Cu Kα radiation (λ = 1.54056 Å); 40 kV and 40 mA; scan speed 8°/min; step size 0.05°, 2θ = 5–40°.

### Single-Crystal X-ray Data Collection and Structure Determination

Crystal structures were determined by single-crystal X-ray diffraction (SCXRD) with either Cu Kα (λ = 1.5418 Å) radiation or Mo Kα (λ = 0.71073 Å) radiation and a Bruker D8 Quest fixed-chi diffractometer equipped with a Photon 100 detector and the nitrogen-flow Oxford Cryosystem attachment. Unit cell determination, data reduction, and absorption correction (multiscan method) were conducted using the Bruker APEX3 suite with implemented SADABS software.^51^ Structures were solved using SHELXT and refined using SHELXL contained in Olex2.^52^ Reflection data for the nonhydrogen atoms were refined anisotropically. All hydrogen atoms bonded to carbon (on phenyl rings, methanol, TMA, TEA, TPA, and TPA cations) were placed geometrically and refined using a riding model with isotropic thermal parameters: *U*_iso_(H) = 1.5*U*_eq_(−CH_3_), *U*_iso_(H) = 1.2*U*_eq_(−CH), *U*_iso_(H) = 1.2*U*_eq_(−CH_2_). Hydrogen atoms on water (AFIX 5) and methanol (AFIX 147) were calculated geometrically and refined using a riding model with isotropic thermal parameter: *U*_iso_(H) = 1.5*U*_eq_(−OH). The hydrogen atoms of phenolic hydroxyl groups were located from electron density difference maps and included in the refinement process using a riding model with *U*_iso_(H) = 1.2*U*_eq_(−OH). Single-crystal data are presented in Table 1.

**Table 1:** Crystallographic Data and Structure Refinement Parameters for the ICCs Reported Herein

	PHNTMA	PHNKOH	IPPTPA	IPPTBA	IPPKOH
formula	C_48_H_75_N_3_O_9_	C_24_H_23_KO_4_	C_87_H_138_N_2_O_7_	C_43_H_73_NO_4_	C_36_H_47_KO_4_
neutral:anion:cation	1:1:1:H_2_O	3:1:1	5:2:2	2:1:1:H_2_O	3:1:1
crystal system	Monoclinic	Monoclinic	Triclinic	Monoclinic	Monoclinic
space group	**P**2_1_/*c*	**Pbca**	*P*1̅	**P**2_1_/**c**	**P**2_1_/**n**
*a* (Å)	18.5854(4)	7.7043 (2)	9.6610(2)	16.6149(12)	7.7087(2)
*b* (Å)	16.2111(3)	22.5580(6)	16.2003(3)	16.6917(11)	26.3556(6)
*c* (Å)	18.3532(3)	25.0836(6)	27.6347(5)	16.3200(13)	16.9093(4)
α (deg)	90	90	100.579(1)	90	90
β (deg)	119.245(10)	90	98.943(1)	111.505(5)	91.120(2)
γ (deg)	90	90	90.087(1)	90	90
*V* (Å^3^)	4824.82(16)	4359.37(19)	4197.92(14)	4211.0(6)	3434.76(14)
*Z*, *Z*′	4, 1	8, 1	2, 1	4, 1	4, 1
*T* (K)	150	150	150	120	150
*R*_1_	0.0511	0.0364	0.0662	0.0804	0.0571
*wR*_2_	0.1492	0.0942	0.1881	0.2415	0.1512
Goff	1.035	1.019	1.051	1.056	1.038
CCDC#	2155483	2155484	2155485	2155486	2155487

	MOPTBA	PGNTMA	PGNTEA	RESTMA
formula	C_44_H_67_NO_8_	C_48_H_73_N_3_O_20_	C_21_H_35_NO_7_	C_32_H_46_N_2_O_8_
neutral:anion:cation	3:1:1	3:3:3:2H_2_O	1:1:1:CH_3_OH	1:1:1
crystal system	triclinic	monoclinic	monoclinic	monoclinic
space group	*P*1̅	**P**2_1_/**n**	*Pc*	**P**2_1_/**c**
*a* (Å)	12.6739(2)	8.9869(1)	8.0556(4)	14.8364(4)
*b* (Å)	16.2081(3)	39.1608(6)	7.9806(4)	12.1427(4)
*c* (Å)	21.2987(3)	14.8299(2)	16.9467(9)	17.7886(4)
α (deg)	79.689(1)	90	90	90
β (deg)	77.183(1)	101.961(1)	90.510(2)	100.267(2)
γ (deg)	89.586(1)	90	90	90
*V* (Å^3^)	4194.86(12)	5105.84(12)	1089.44(4)	3153.37(14)
*Z*, *Z*′	4, 2	4, 1	2, 1	4, 1
T (K)	130	144	150	150
*R*_1_	0.0452	0.0477	0.0279	0.0387
*wR*_2_	0.1216	0.1672	0.0721	0.1074
Goff	1.021	1.185	1.052	1.032
CCDC#	2155488	2155489	2155490	2155491

### CSD Analysis

**Figure 1:**
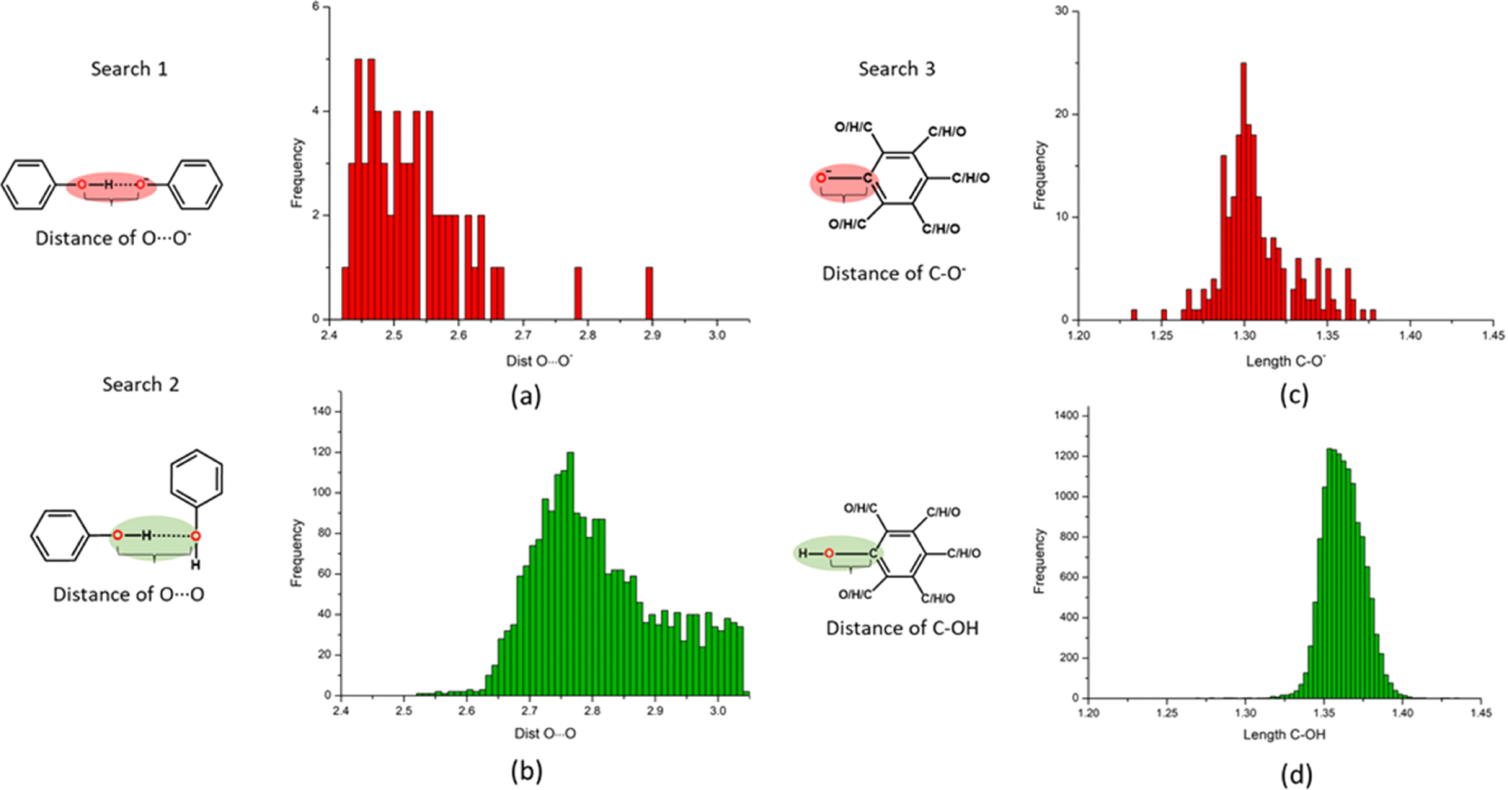
Histograms representing the distance distributions of (a) O···O^–^ between phenol and phenolate, (b) O···O between phenol and phenol, (c) C–O bond in PhOH, and (d) C–O^–^ bond in PhO^–^.

A CSD survey (ConQuest version 2020.3.0 with May 2021 update, search parameters: 3D coordinates present; only organics; *R* factor ≤ 0.05, no disorder and single-crystal structure only) was conducted to find crystal structures that contain PhOH···PhO^–^ interactions. The following parameters were addressed by the search: (1) number of structures that exhibit phenol–phenolate interactions excluding single-component structures, salts, and zwitterions; (2) number of structures that form a PhOH···PhOH supramolecular homosynthon; (3) number of structures that contain a phenol group or a phenolate anion with the restriction that only C, H, and O were considered as substituents on the phenyl ring (excluding molecules containing strong electron-withdrawing or -donating groups such as Cl and −NH_2_, which were not surveyed). The distance distribution of phenol–phenolate and phenol–phenol hydrogen bonds and the bond length distribution of the C–O bond of the phenol or phenolate group were plotted based on data from Searches 1, 2, and 3. The schematic structures used in searches 1, 2, and 3 are presented in Figure 1.

### Computational Methods

The intermolecular interaction energies of charge-assisted phenol–phenolate hydrogen bonds and neutral phenol–phenol hydrogen bonds were calculated using monomer wavefunctions at the B3LYP/6-31G(d,p) level in the CrystalExplorer program package followed by geometry optimization carried out using the CASTEP module with GGA-type PBE functional contained in Materials Studio 8.0. The total interaction energy was partitioned into electronic (E_ele), polarization (E_pol), dispersion (E_dis), and repulsion (E_rel) components.^53^ The total energy and four key energy terms of each nonequivalent hydrogen bond involved in all ionic cocrystals reported here are presented in Table S1 in the Supporting Information.

## Results and Discussion

### CSD Analysis of the Phenol–Phenolate Supramolecular Heterosynthon

Our CSD search retrieved 156 entries in which PhOH···PhO^–^ interactions are present. Of these entries, 69 (44.2%) are single-component compounds with phenol and phenolate moieties in the same molecule; 30 entries (19.2%) are salts, and 15 (9.6%) are zwitterionic cocrystals or solvates composed of a zwitterion and a neutral molecule; 42 (26.9%) structures meet the definition of an ICC that contains a phenol molecule, phenolate anion, and cation. However, of these 42 cocrystals, only one paper^54^ classifies them as cocrystals (refcode: JICKIS).

### CSD Analysis of Phenol–Phenolate Hydrogen-Bond Distance Distribution (O–H···O^–^)

The PhOH···PhO^–^ O···O^–^ bond distance determined from 42 ICC structures ranged from 2.4195(52)–2.6599(24) Å with an average of 2.528 ± 0.08Å. A histogram of the O···O^–^ distance distribution for PhOH···PhO^–^ interactions is presented in Figure 1a.

### CSD Analysis of Phenol–Phenol Hydrogen-Bond Distance Distribution (O–H···O)

O–H···O bond distances of PhOH···PhOH interactions determined from search 2 ranged from 2.5218(28)–3.0381(45) Å, averaging 2.812 ± 0.103 Å. A histogram of the O–H···O hydrogen-bond distance distribution for PhOH···PhOH interactions is presented in Figure 1b.

### CSD Analysis of C–O Bond Length Distribution in Phenols vs C–O^–^ in Phenolates

CSD search 3 addressed the C–O bond length in phenols vs phenolates. The CSD was found to contain 8277 and 192 entries for phenols and phenolates, respectively. Figure 1c,d reveals that C–OH bonds range from 1.2562(29) to 1.5082(37) Å with a mean of 1.362 ± 0.013 Å, whereas C–O^–^ bonds range from 1.2341(27) to 1.3776(95) Å with a mean of 1.307 (0.023) Å. The distribution plots in Figure 1 reveal very few outliers and C–O bond length can therefore be indicative of protonation.

### Description of Crystal Structures

Hydrogen bonds were found to be present in each of the nine novel ICCs reported herein: PhO^–^···PhOH; PhOH···PhOH; PhO^–^···H_2_O/MeOH; PhOH···H_2_O/MeOH. Tetramethylammonium (TMA), tetraethylammonium (TEA), tetrapropylammonium (TPA), and tetrabutylammonium (TBA) cations exhibit no strong hydrogen bonds. Rather, coulombic forces occur with phenolate anions. The neutral or ionic nature of phenolic groups in the nine ICCs was addressed through proton location^55,56^ from difference Fourier map inspection and C–O bond lengths. Table S1 lists the C–O bond lengths in the neutral (C–OH) and deprotonated (C–O^–^) moieties.

**Figure 2:**
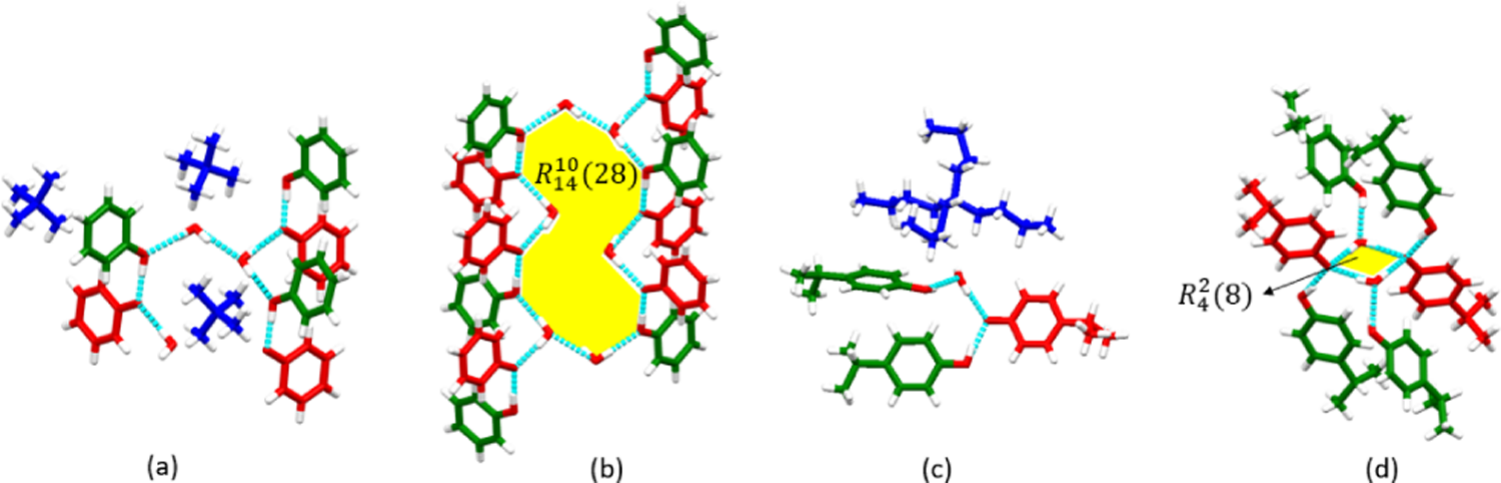
(a) Asymmetric unit and (b) ring motif in PHNTMA. (c) Asymmetric unit and (d) ring motif in IPPTBA. TMA and TBA cations are omitted for clarity in (b) and (d). Molecules, anions, and cations are colored green, red, and blue, respectively.

PHNTMA, IPPTPA, IPPTBA, and MOPTBA formed discrete adducts. PHNTMA and IPPTBA crystallized as hydrates sustained by PhOH···PhO^–^ and PhO^–^···H_2_O hydrogen bonds. PHNTMA crystallized in the space group **P**2_1_/*c*. The asymmetric unit of PHNTMA contains three phenol molecules, three phenolate anions, three TMA cations, and three water molecules in the ratio of 1:1:1:1 (Figure 2a). Four phenol molecules, four phenolate anions, and six water molecules participate in a ring motif with the graph set notation *R*_14_^10^(28) (Figure 2b). The ring motif and two phenol groups located at opposite ends of the ring afford a discrete adduct. Phenol molecules hydrogen-bond with water molecules while also acting as hydrogen-bond acceptors. Three charge-assisted PhOH···PhO^–^ hydrogen bonds within the discrete unit exhibit O···O^–^ bond distances of 2.438(2), 2.434(2), and 2.466(2) Å. At the (100) plane, adjacent discrete units are arranged vertically and interact via TMA cations. The discrete units align along the *a*-axis.

IPPTBA crystallized as a 2:1:1:1 ICC of 4-isopropylphenol, 4-isopropylphenolate, TBA, and water in the space group **P**2_1_/**c** (Figure 2c). Two 4-isopropylphenolate and two water molecules constitute an *R*_4_^2^(8) hydrogen-bonded ring with each molecule interacting with an extra IPP molecule outside the ring to generate a discrete unit (Figure 2d). The ring motif contains one PhOH···PhO^–^ hydrogen bond with O···O^–^ = 2.528(3) Å. IPP serves as a hydrogen-bond donor and is hydrogen-bonded to a water molecule in each motif. The discrete units are connected by TBA cations and aligned along the *a*-axis.

**Figure 3:**
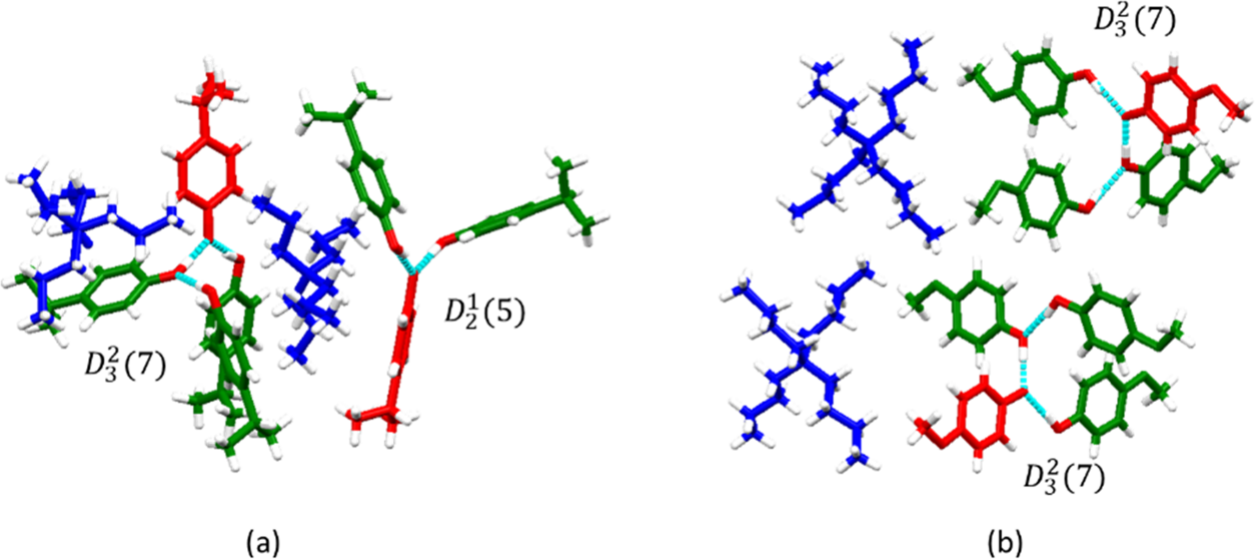
Discrete supramolecular structures in (a) IPPTPA and (b) MOPTBA. Molecules, anions, and cations are colored green, red, and blue, respectively.

IPPTPA and MOPTBA both crystallized in space group *P*1̅ with one (*Z*′ = 1) and two (*Z*′ = 2) formula units in the asymmetric unit, respectively. In both structures, two discrete hydrogen-bonding assemblies exist in each asymmetric unit, forming two different motifs *C*_3_^2^(7) and *C*_2_^1^(5) in IPPTPA (Figure 3a) and two *C*_3_^2^(7) motifs in MOPTBA (Figure 3b). These motifs are sustained by neutral PhOH···PhOH hydrogen bonds and charge-assisted phenol–phenolate hydrogen bonds. Four nonequivalent charge-assisted PhOH···PhO^–^ hydrogen bonds are involved in both structures, respectively (O···O^–^ distance in IPPTPA: 2.427(2) Å, 2.562(2) Å, 2.571(3) Å, 2.589(3) Å; O···O^–^ distances in MOPTBA: 2.5920(16) Å, 2.4413(17) Å, 2.4369(17) Å, 2.6111(17) Å). CH···O interactions and cations link the discrete units.

**Figure 4:**
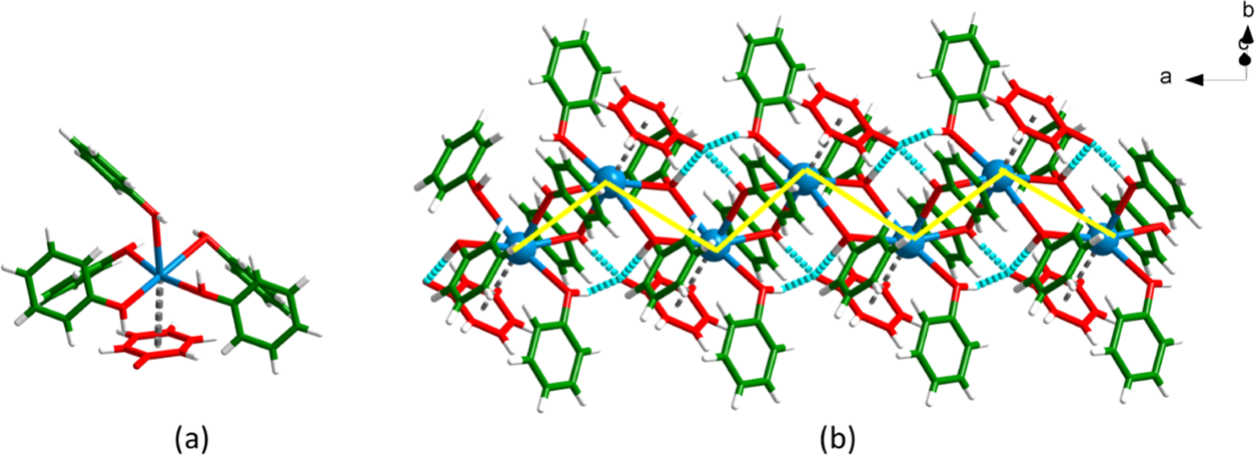
(a) Potassium coordination and (b) one-dimensional coordination polymer in PHNKOH.

PHNKOH and IPPKOH form one-dimensional (1D) coordination polymers. The structure of PHNKOH (3:1:1 ratio of phenol:phenolate:cation, CSD Refcode HERCIQ) and its 2:1:1 variant (CSD Refcode HEQZUY) were previously reported as phenol solvates of potassium phenolate from high-resolution PXRD data by Dinnebier et al.^48^ Herein, single crystals of PHNKOH were grown from MeOH and are isostructural with HERCIQ but with a lower unit cell volume (4359.37 vs 4494.18 Å^3^), presumably due to data collection at 150 K vs room temperature. IPPKOH crystallized in **P**2_1_/**n**. Although they adopt different space groups, PHNKOH and IPPKOH both exhibit asymmetric units comprising three phenol molecules, one potassium cation, and one phenolate anion in a 3:1:1 ratio. Each potassium cation adopts octahedral coordination geometry with five oxygen atoms from phenol molecules (or phenol moieties of IPP), and the sixth coordination site is occupied by the π system of a phenolate anion (Figures 4a and S1a). The 1D coordination polymers in PHNKOH and IPPKOH form zigzag chains (Figures 4b and S1b) that are supported by three nonequivalent charge-assisted hydrogen bonds between each phenolate moiety and three phenol moieties (O···O^–^ in PHNKOH = 2.5276(18) Å, 2.6052(19) Å, 2.6315(19) Å; O···O^–^ in IPPKOH = 2.541(2) Å, 2.617(2) Å, 2.546(3) Å).

**Figure 5:**
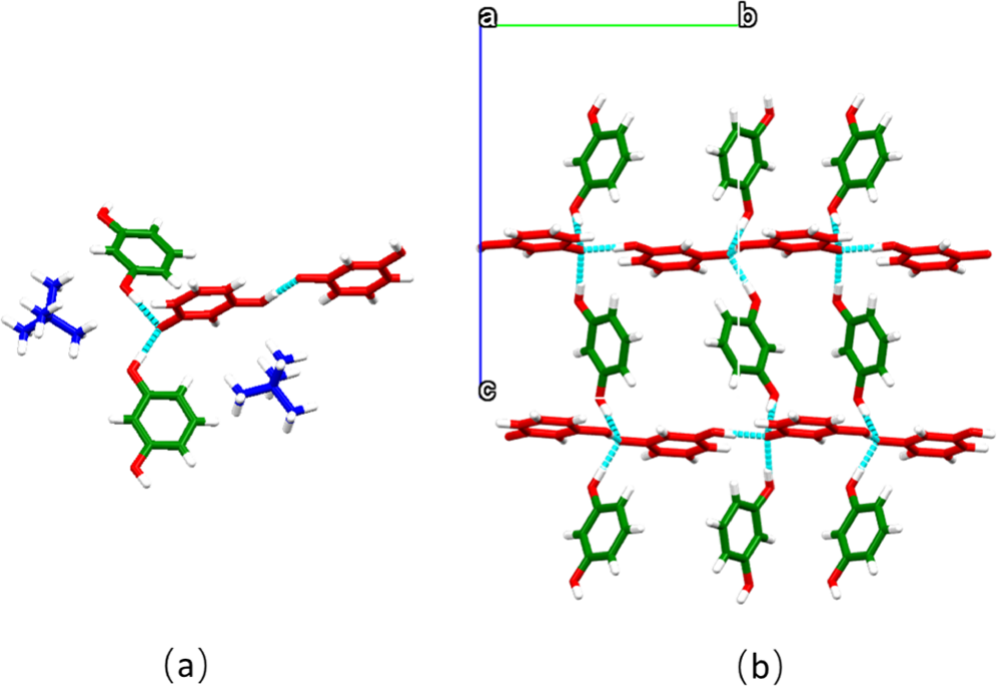
(a) Asymmetric unit and (b) 2D hydrogen-bonding network in RESTMA (TMA cations are omitted for clarity). Molecules, anions, and cations are colored green, red, and blue, respectively.

**RESTMA** is a 1:1:1 ICC of resorcinol, resorcinolate, and TMA (Figure 5a), which crystallized in space group **P**2_1_/**c** as two-dimensional (2D) hydrogen-bonded networks. Deprotonated resorcinol serves as a hydrogen-bond donor and acceptor since only one hydroxyl group is deprotonated. The phenolate moieties of the resorcinolate anion form three charge-assisted PhOH···PhO^–^ hydrogen bonds. The resorcinolate anions interact with each other to form a chain supported by PhOH···PhO^–^ hydrogen bonds. These interactions result in perpendicular chains bridged by resorcinol molecules, resulting in grid-like networks (Figure 5b).

**Figure 6:**
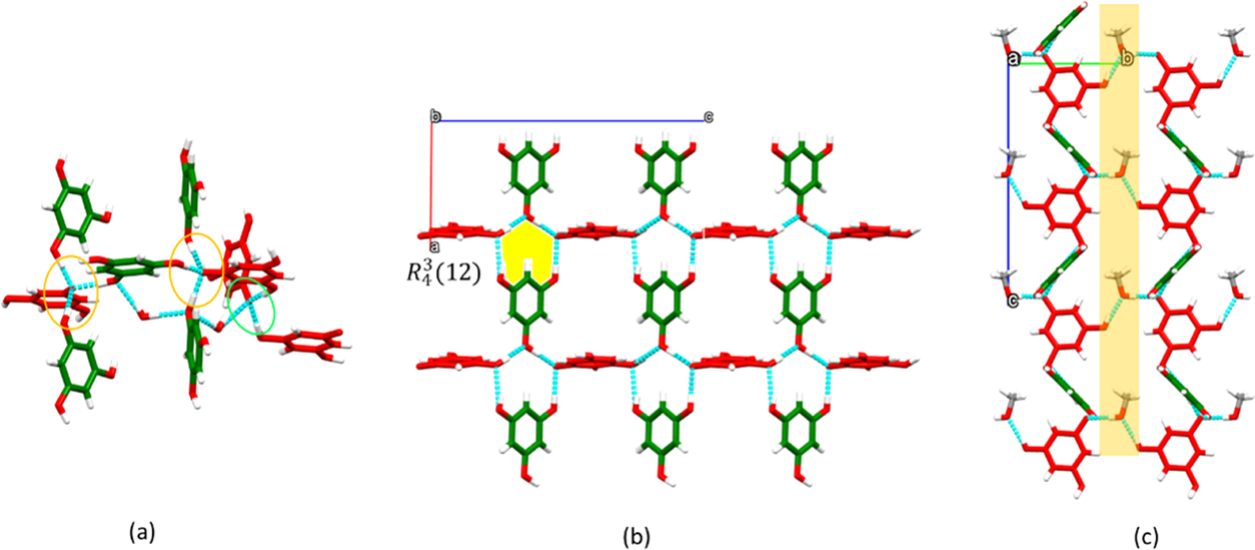
Intermolecular hydrogen-bonding networks in (a) PGNTMA and (b, c) PGNTEA. TMA and TEA cations are omitted for clarity. Molecules and anions are colored green and red, respectively.

PGNTMA and PGNTEA crystallized in the space groups **P**2_1_/**n** and *Pc*, respectively, as 3D networks. In both structures, although there are three possible deprotonation sites, each PGN is monodeprotonated and serves as a hydrogen-bond acceptor and donor. PGNTMA is a hydrate with a 3:3:3:2 ratio of PGN molecules, deprotonated PGN anions, TMA cations, and water molecules. PGNTMA contains three independent deprotonated PGN anions in the asymmetric unit. One phenolate moiety is hydrogen-bonded to two phenolic moieties (PhOH···PhO^–^) and one water molecule (PhO^–^···H_2_O). Two phenolate moieties hydrogen-bond to phenolic groups via PhOH···PhO^–^ hydrogen bonds (Figure 6a). Phenolic groups also form PhOH···H_2_O interactions, facilitating the formation of a 3D hydrogen-bonded network. PGNTEA is a methanol solvate ICC with one PGN molecule, one deprotonated PGN anion, one TEA cation, and one MeOH molecule in the asymmetric unit. The phenolate and phenol moieties are hydrogen-bonded through an *R*_4_^3^(12) ring motif to expand into a ladder-like plane composed of rails (red deprotonated PGN chains in Figure 5b) and rungs (green neutral PGN chain in Figure 6b) sustained by PhOH···PhO^–^ and PhOH···PhOH hydrogen bonds. Adjacent parallel planes are connected by MeOH molecules, affording three-dimensional (3D) hydrogen-bonded networks through PhO^–^···MeOH and PhOH···MeOH hydrogen bonds, respectively (Figure 6c).

As detailed above, charge-assisted PhOH···PhO^–^ hydrogen bonds were observed in each of the ICCs synthesized herein. Four ICCs (IPPTPA, MOPTBA, PGNTMA, PGNTEA) also feature PhOH···PhOH supramolecular homosynthons. The two most common motifs observed are those in which a phenolate moiety interacts with two (in IPPTPA, MOPTBA, PHNTMA) or three hydroxyl donors (in RESTMA, IPPKOH, IPPTBA, PGNTEA, PGNTMA, PHNKOH). Phenols are generally more acidic than aliphatic alcohols but less acidic than most carboxylic acids. This study presents a strategy to form ICCs of phenols and their conjugate bases by PhOH···PhO^–^ interactions to drive and sustain crystal packing. It is a well-recognized rule of thumb for salt and cocrystal screening that if Δp*K*_a_ (Δp*K*_a_ = p*K*_a_ (base)-pK_a_ (acid)) is >3, then a salt is formed and that if Δp*K*_a_ is <0, then a cocrystal is favored.^57^ In this work, Δp*K*_a_ is >3 for all bases used, and as such, proton transfer was anticipated even though proton transfer could have been affected by factors such as solvent and the composition of starting materials. In this study, several solvent systems were used for ICC screening. Except for PHNTMA, only the use of MeOH or H_2_O as solvent afforded high-quality single crystals suitable for SCXRD data collection. Poor quality crystals or gel-like phases were produced using ethanol, 2-propanol, or acetone.

### Hydrogen-Bond Strengths

**Figure 7:**
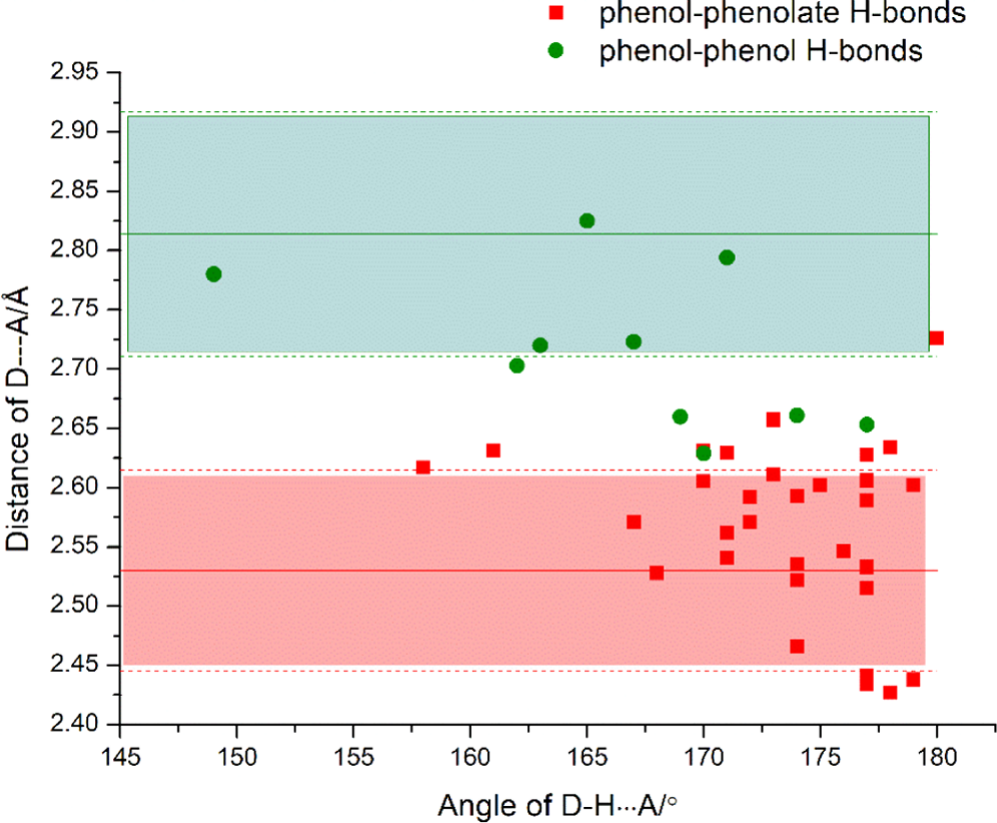
Plot of selected hydrogen bond distances in ICCs (red zone = the O···O^–^ distance in PhOH···PhO^–^ interactions from search 1; green zone = O···O distance in PhOH···PhOH hydrogen bonds from search 2; red squares and green circles are PhOH···PhO^–^ and PhOH···PhOH hydrogen bonds observed in the ICCs reported herein).

**Figure 8:**
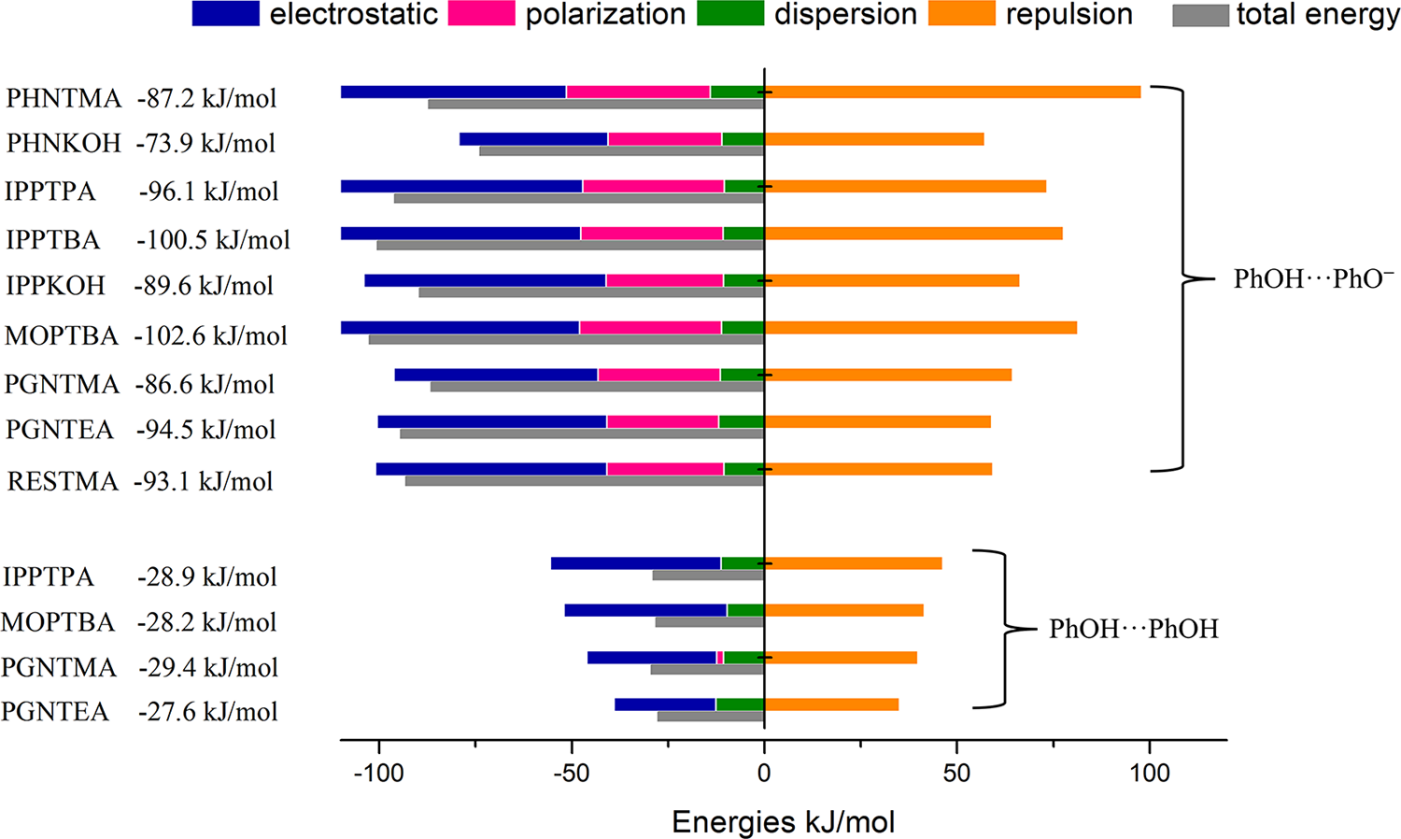
Contributions of electrostatic, polarization, dispersion, and repulsion interactions to the total energies of selected hydrogen bonds (total energies are shown on the left). The polarization term is often negligible for neutral hydrogen bonds as its value is less than dispersion values.

Selected hydrogen bond lengths for PhOH···PhO^–^ and PhOH···PhOH interactions in the nine new ICCs reported herein are tabulated in Table S2 and presented as a scatter plot in Figure 7. Gas-phase hydrogen bond strengths and contributions to the total energies from electrostatic, polarization, dispersion, and repulsion interactions for selected hydrogen bonds were calculated (Figure 8). In most of the ICCs reported herein, multiple types of hydrogen bonds were observed in terms of the directionality, distance, and angle in the refined crystal structures. In these cases, interaction energies of all nonsymmetrically equivalent possibilities were calculated (see Table S2), and the average strength is presented in Figure 8.

As shown by the scatter plot of Figure 7, the PhOH···PhO^–^ and PhOH···PhOH hydrogen-bond distances in the nine novel ICCs fall in the “red zone” and “green zone”, respectively. These distance zones are based upon O···O^–^ distance ranges in PhOH···PhO^–^ and PhOH···PhOH hydrogen bonds (Å), respectively, determined from crystal structures archived in the CSD. PhOH···PhO^–^ hydrogen bonds were found to exhibit relatively short distances (2.427(2)–2.658(2) Å) and angles nearing 180°, whereas PhOH···PhOH hydrogen-bond distances were longer (2.825(3)–2.6289(19) Å) and angles were more acute (149(5)–177(8)°). Some charge-assisted PhOH···PhO^–^ hydrogen bonds have relatively long O···O^–^ distances or small angle values (2.6315(19) Å, 161(3)° in PHNKOH; 2.617(2) Å, 158(8)° in IPPKOH; 2.726(3) Å, 180(7)° in PGNTEA). These parameters are likely affected by the different crystal packing environment in each crystal structure. Nevertheless, as expected, charge-assisted PhOH···PhO^–^ hydrogen bonds were found to be consistently shorter than PhOH···PhOH hydrogen bonds. Further, as presented in Figure 8, PhOH···PhO^–^ interactions were calculated to offer ca. 3 times the energy of PhOH···PhOH interactions, with average energies of 91.6 ± 8.1 and 28.53 ± 0.67 kJ/mol, respectively. As also detailed in Figure 8, the electrostatic contributions were calculated to be the dominant contribution to PhOH···PhO^–^ and PhOH···PhOH hydrogen-bond strength. Specifically, for PhOH···PhOH interactions, the electrostatic contribution comes from dipole···dipole interactions, whereas for PhOH···PhO^–^ interactions, the phenolate oxygen and phenolic hydrogen are key to the electrostatic contribution. The greater electrostatic energies in PhOH···PhO^–^ can therefore be attributed to coulombic forces. Polarization contributes to total energy for PhOH···PhO^–^, while it is negligible for PhOH···PhOH. A small dispersion contribution was calculated for all hydrogen bonds. Repulsion forces for PhOH···PhO^–^ were consistently calculated to be greater than for PhOH···PhOH hydrogen bonds. Overall, although repulsive forces for the charge-assisted hydrogen bonds are relatively high, the increase is more than offset by increases in permanent charge and electronic and polarization forces. These high intermolecular interaction energies and desirable hydrogen-bond geometry features validate that charge-assisted PhOH···PhO^–^ hydrogen bonds are a strong and reliable interaction to enable the formation of ionic cocrystals.

## Conclusions

Phenol groups are found in 8.7% of approved small-molecule drugs in the DrugBank database and in 10.1% of bioactive single-component compounds deposited in the CSD. Our CSD survey revealed that the PhOH···PhO^–^ supramolecular heterosynthon is understudied in the context of crystal engineering of ICCs. To address this issue, nine novel ICCs involving phenol and phenol-substituted compounds were formed by reaction with bases and their crystal structures were determined by SCXRD. Analysis of the resulting crystal structures revealed that all nine ICCs are sustained by the phenol–phenolate supramolecular heterosynthon with O···O^–^ distances ranging from 2.427(2) to 2.658(2) Å. A computational study validated the robustness of the charge-assisted PhOH···PhO^–^ supramolecular heterosynthon vs. the phenol–phenol supramolecular homosynthon from an energetic perspective. Based on these results, we consider that the PhOH···PhO^–^ supramolecular heterosynthon is suitable for the reliable formation of ICCs of phenolic compounds, which are prevalent in drug molecules and nutraceuticals. That phenols form ICCs with their conjugate bases is important from a pharmaceutical perspective as it means that the mass of a dosage form can be close to that of the parent phenol. Future studies will focus on pharmaceutical and nutraceutical cocrystals, their physicochemical properties, and polymorphism.

## Supporting Information Available

Coordination details and packing in IPPKOH; distance, angles, and energies of selected hydrogen bonds in all ICCs; length of C–O bond in all ICCs; and PXRD comparison of calculated and experimental patterns (PDF)
