# Supplementary material for: Crystal Engineering of Ionic Cocrystals Sustained by the Phenol–Phenolate Supramolecular Heterosynthon
Source: Cryst Growth Des. 2022 Jun 21;22(7):4582–91. doi: 10.1021/acs.cgd.2c00471 (PMC9347308; doi:10.1021/acs.cgd.2c00471)
Supplement: Supplementary file 1 — cg2c00471_si_001.pdf [file cg2c00471_si_001.pdf]

Supporting Information

Crystal Engineering of Ionic Cocrystals Sustained by the Phenol-Phenolate Supramolecular Heterosynthon

*Shasha Jin[a], Rana Sanii[a], Bai-Qiao Song[a] and Michael J. Zaworotko[a]**

[a]Department of Chemical Sciences and Bernal Institute, University of Limerick, Co, Limerick, V94 T9PX, Ireland

*Email: xtal@ul.ie

TABLE OF CONTENTS

CSD search parameters --2

IPPKOH polymer figures -- 3

C–O and C–O^- bond lengths --4

Hydrogen bonds parameters ---5,6

PXRD patterns ---7,8

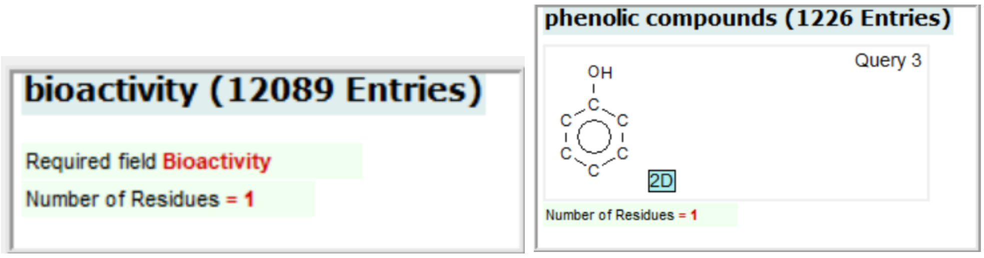

Scheme S1 CSD search (ConQuest version 2020.3.0 with Sep 2021 update) for all bioactive single-component compounds and in which phenolic compounds (search parameters: 3D-coordinates present; only organics; and single crystal structure only).

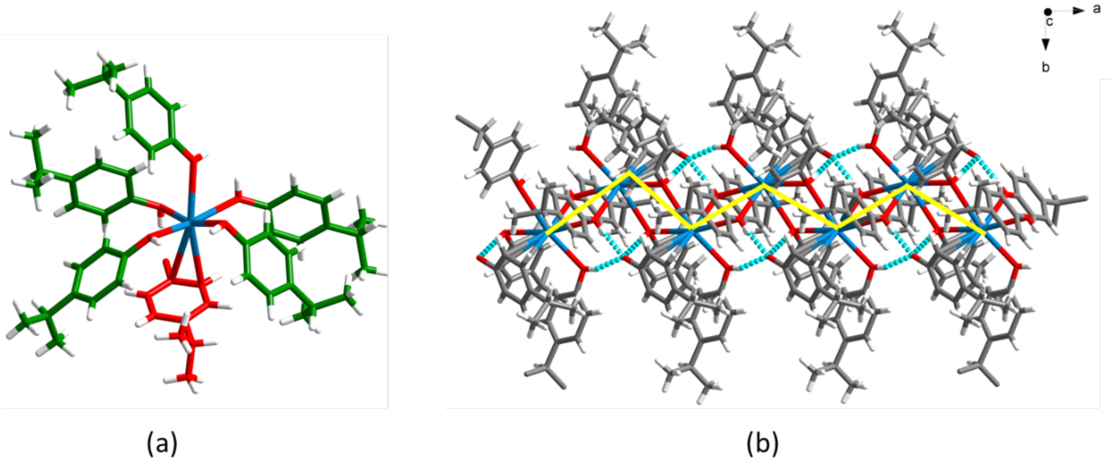

Figure S1 (a)potassium coordination in IPPKOH (red: deprotonated IPP, green: neutral IPP, blue: potassium) (b)zig-zag chain in IPPKOH

Table S1 Length of C–O bond in neutral species and C–O^- bond in anion species

ICCs	d (C–O^-)/Å	d (C–O)/Å	ICCs	d (C–O^-)/Å	d (C–O)/Å
PHNTMA	1.325<br>1.339<br>1.336	1.341<br>1.342<br>1.345	MOPTBA	1.345<br>1.348	1.353<br>1.356<br>1.363<br>1.363<br>1.359<br>1.356
PHNKOH	1.351	1.369<br>1.369<br>1.377	PGNTMA	1.346<br>1.356<br>1.359	1.377<br>1.360<br>1.375<br>1.360<br>1.373<br>1.366<br>1.380<br>1.354<br>1.363<br>1.364<br>1.375<br>1.371<br>1.374<br>1.368<br>1.382
IPPTPA	1.343<br>1.319	1.348<br>1.358<br>1.365<br>1.364<br>1.361	PGNTEA	1.349	1.368<br>1.387<br>1.369<br>1.365<br>1.364
IPPTBA	1.345	1.360<br>1.365	RESTMA	1.349<br>1.346	1.356<br>1.353<br>1.354<br>1.362<br>1.365<br>1.362
IPPKOH	1.348	1.372<br>1.373<br>1.377			

Table S2 Distance and parameters of charged-assisted hydrogen bonds and neutral hydrogen bonds for all the ionic cocrystals reported here (marked red values are interaction energies of neutral H bond but involving a charge, which is located at other deprotonated hydroxyl group on the phenyl ring of PGN; marked blue values are interaction energies of neutral H bond but involving two charge)

Ionic cocrystals	d (D–H)/Å	d (H···A)/Å	D (D··· A)/Å	θ/°	E_ele kJ·mol^{-1}	E_pol kJ·mol^{-1}	E_dis kJ·mol^{-1}	E_rep kJ·mol^{-1}	E_tot kJ·mol^{-1}
	Charge-assisted H bond of phenol-phenolate								
PHNTMA	0.94(3)	1.50(3)	2.438(2)	179(2)	-145.7	-52.3	-14.5	102.2	-110.3
	0.95(4)	1.52(4)	2.466(2)	174(3)	-96.9	-49.6	-13.8	86.0	-74.2
	0.89(4)	1.55(4)	2.434(2)	177(5)	-116.1	-52.5	-14.2	105.6	-77.2
PHNKOH	0.87(3)	1.67(3)	2.5276(18)	168(3)	-95.7	-46.5	-12.0	73.4	-80.8
	0.90(3)	1.76(3)	2.6315(19)	161(3)	-67.6	-37.1	-8.0	43.9	-68.8
	0.94(3)	1.68(3)	2.6052(19)	170(3)	-74.8	-38.3	-13.2	54.3	-72.2
IPPTPA	0.94(4)	1.49(4)	2.427(2)	178(8)	-132.8	-53.1	-12.9	108.3	-90.7
	1.06(6)	1.51(6)	2.562(2)	171(5)	-99.3	-46.1	-9.4	60.6	-94.2
	0.92(6)	1.67(6)	2.571(3)	167(5)	-107.8	-43.7	-8.7	61.9	-98.4
	0.95(6)	1.65(6)	2.589(3)	177(7)	-107.3	-45.7	-10.6	62.6	-101.1
IPPTBA	0.92(4)	1.62(5)	2.528(3)	168(5)	-119.7	-47.7	-10.7	77.6	-100.5
IPPKOH	0.93(7)	1.62(8)	2.541(2)	171(6)	-115.0	-45.6	-12.5	79.2	-94.3
	0.89(7)	1.66(7)	2.546(3)	176(7)	-107.0	-40.3	-10.5	69.0	-88.7
	0.85(7)	1.81(7)	2.617(2)	158(8)	-90.2	-37.5	-9.0	50.9	-85.8
MOPTBA	0.94(3)	1.66(3)	2.5920(16)	172(3)	-114.7	-44.5	-9.1	63.7	-104.6
	0.96(2)	1.48(2)	2.4413(17)	177(2)	-140.2	-50.8	-12.5	100.5	-103.0
	0.94(2)	1.49(2)	2.4369(17)	177(3)	-140.2	-52.2	-13.6	106.4	-99.7
	0.95(3)	1.66(3)	2.6111(17)	173(2)	-104.0	-44.7	-9.5	55.0	-103.2
PGNTMA	0.97(6)	1.67(6)	2.629(2)	171(5)	-97.3	-38.6	-12.5	51.7	-96.8
	1.04(6)	1.54(6)	2.571(2)	172(4)	-111.9	-44.9	-14.7	72.5	-99.2

	0.99(6)	1.55(6)	2.535(3)	174(5)	-124.4	-47.1	-8.7	71.6	-108.7
	0.95(6)	1.71(6)	2.658(2)	173(5)	-46.7	-42.8	-8.3	46.8	-51.0
	1.04(5)	1.50(5)	2.533(2)	177(5)	-103.0	-44.3	-12.5	74.7	-85.2
	0.95(5)	1.69(6)	2.634(2)	178(8)	-91.5	-39.1	-11.1	53.7	-88.0
	1.00(6)	1.52(5)	2.522(2)	174(6)	-102.0	-48.1	-13.6	84.0	-79.8
	0.92(6)	1.69(6)	2.606(2)	177(7)	-92.7	-41.2	-10.3	60.3	-83.9
PGNTEA	0.83(6)	1.90(6)	2.726(3)	180(7)	-81.9	-36.3	-10.6	41.2	-87.7
	0.98(5)	1.54(5)	2.515(2)	177(6)	-119.2	-45.7	-13.2	76.8	-101.3
RESTMA	0.88 (3)	1.78 (3)	2.6566 (15)	173 (2)	-80.6	-38.9	-9.6	48.0	-81.1
	0.95 (2)	1.65 (2)	2.6016 (14)	175 (2)	-95.3	-39.7	-12.0	58.8	-88.4
	0.94 (3)	1.66 (3)	2.6023 (14)	179 (4)	-100.1	-41.8	-12.7	61.9	-92.8
	0.91 (3)	1.73 (3)	2.6312 (15)	170 (2)	-80.3	-38.6	-10.0	52.0	-76.9
	0.96 (2)	1.64 (2)	2.5925 (14)	174 (3)	-137.2	-44.0	-9.1	71.7	-118.7
	0.93 (3)	1.70 (3)	2.6278 (16)	177 (2)	-112.3	-42.4	-9.7	63.5	-100.9
Neutral H-bond of phenol-phenol									
IPPTPA	0.86(7)	1.79(6)	2.653(3)	177(8)	-55.6	-8.3	-11.2	46.4	-28.9
MOPTBA	0.95(3)	1.73(3)	2.6597(18)	169(2)	-50.9	-7.6	-9.8	40.0	-28.3
	0.89(3)	1.75(3)	2.6289(19)	170(2)	-53.3	-8.0	-9.7	43.0	-28.1
PGNTMA	0.90(4)	1.84(4)	2.723(3)	167(4)	-37.5	-16.5	-9.5	42.7	-20.8
	0.98(5)	1.69(6)	2.661(3)	174(6)	-61.7	-9.0	-10.8	53.4	-28.2
	0.80(6)	1.93(6)	2.703(2)	162(6)	-45.1	-16.7	-10.5	41.7	-30.8
	0.89(6)	1.98(6)	2.780(2)	149(5)	-33.0	-13.8	-9.1	25.8	-30.1
	0.85(6)	1.95(6)	2.794(2)	171(6)	-53.5	-6.1	-13.2	35.7	-37.2
PGNTEA	0.89(5)	1.85(5)	2.720(2)	163(5)	-57.6	-8.1	-14.1	43.9	-36.0
	0.82(6)	2.02(6)	2.825(3)	165(5)	-20.4	-14.1	-11.1	26.2	-19.3

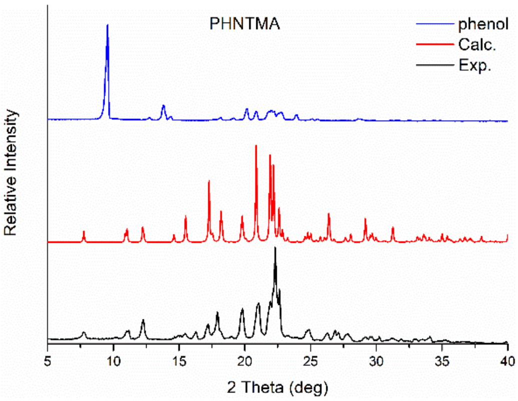
PHNTMA
phenol
Calc.
Exp.
Relative Intensity
5 10 15 20 25 30 35 40
2 Theta (deg)

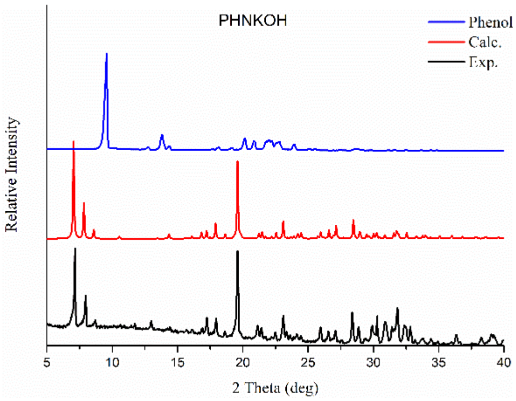
PHNKOH
Phenol
Calc.
Exp.
Relative Intensity
5 10 15 20 25 30 35 40
2 Theta (deg)

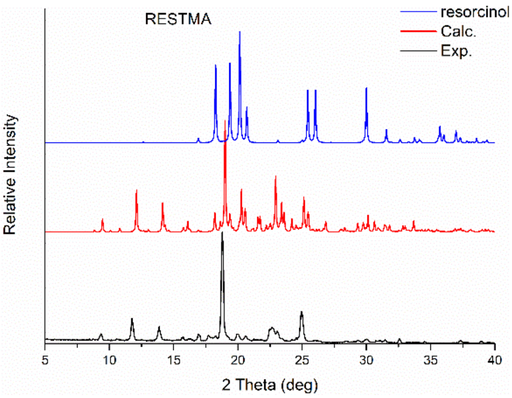
RESTMA
resorcinol
Calc.
Exp.
Relative Intensity
5 10 15 20 25 30 35 40
2 Theta (deg)

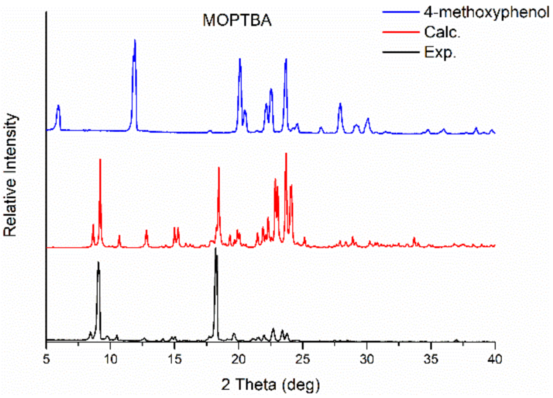
MOPTBA
4-methoxyphenol
Calc.
Exp.
Relative Intensity
5 10 15 20 25 30 35 40
2 Theta (deg)

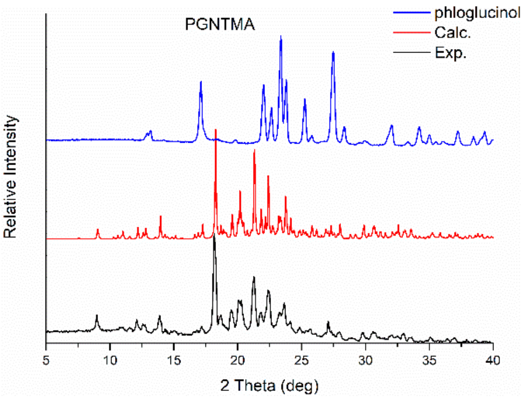
PGNTMA
phloglucinol
Calc.
Exp.
Relative Intensity
5 10 15 20 25 30 35 40
2 Theta (deg)

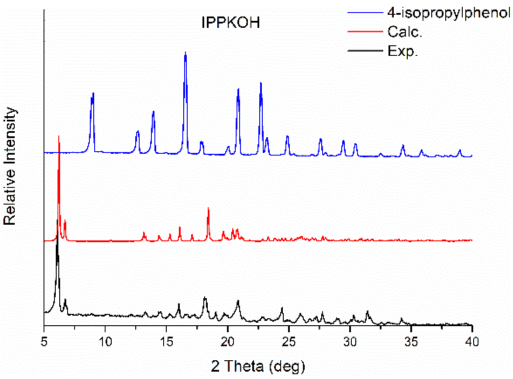
IPPKOH
4-isopropylphenol
Calc.
Exp.
Relative Intensity
5 10 15 20 25 30 35 40
2 Theta (deg)

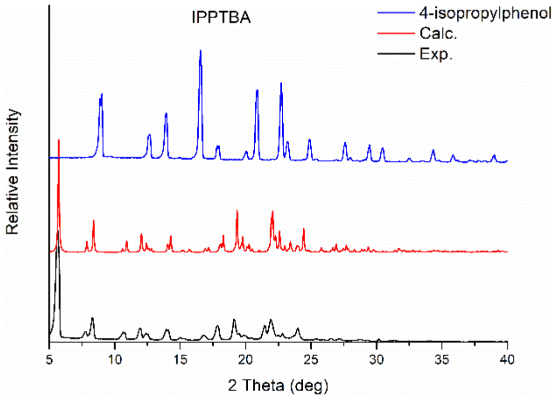

Figure S2 Calculated PXRD from single crystals of ionic cocrystals compared with those as synthesized.
